# Tailored Anterior Clinoidectomy: Beyond the Intradural and Extradural Concepts

**DOI:** 10.7759/cureus.14874

**Published:** 2021-05-06

**Authors:** Messias Gonçalves Pacheco Junior, José Orlando de Melo Junior, Marcus André Acioly, Raíssa Mansilla Cabrera Rodrigues, Bruno Lima Pessôa, Rafael A Fernandes, José Alberto Landeiro

**Affiliations:** 1 Neurosurgery, Federal Fluminense University, Niterói, BRA; 2 Neurosurgery, Paulo Niemeyer State Brain Institute, Rio de Janeiro, BRA; 3 Neurosurgery, Hospital Universitario Antonio Pedro, Niterói, BRA

**Keywords:** anterior clinoid process, extradural clinoidectomy, intradural clinoidectomy, tailored anterior clinoidectomy, skull base surgery

## Abstract

Anterior clinoidectomy (AC) is a key microsurgical step for the safe and successful management of parasellar pathologies that involve the anterior clinoid process (ACP) and the optic canal. Traditionally, extra and intradural ACs are performed separately according to the surgeon's experience or preference. The objective is to present and discuss the tailored AC concept through illustrative cases. We conducted a retrospective record review of three patients who underwent AC as a surgical step for the treatment of parasellar pathologies that involve the ACP and optic canal. A review of the relevant literature on AC was performed in the PubMed, LILACS, and SciELO databases.

In all three cases, the pterional craniotomy was the preferred approach for AC. Case 1, a 47-year-old female patient with type III anterior clinoidal meningioma, underwent a tailored intradural technique (optic canal unroofing) with total tumor resection and complete visual recovery. Case 2, a 63-year-old female patient with a complex type II anterior clinoidal meningioma with extensive hyperostosis of the ACP, underwent a hybrid AC technique with complete removal of the tumor and visual improvement. Case 3, a 62-year-old female, underwent a tailored intradural AC for clipping an incidental carotid-ophthalmic aneurysm.

Tailored AC aims to provide adequate exposure with less risk of neurovascular injury, allowing enough space to safely treat parasellar lesions. The type, size, and location of the lesion, as well as the surgeon’s experience, should always be considered for surgical planning.

## Introduction

The anterior clinoid process (ACP) is a triangular-shaped process of the sphenoid bone, which is a continuation of the lesser sphenoid wing and is medially connected to the sphenoid body by the optic strut and roof of the optic canal [1]. ACP relationships comprise the oculomotor nerve inferiorly, the internal carotid artery (ICA) inferomedially, and the optic nerve medially. Furthermore, the optic strut separates the optic canal from the superior orbital fissure [2].

Anterior clinoidectomy (AC) is a key microsurgical step for the safe and successful management of paraclinoid aneurysms and parasellar neoplastic lesions, increasing the surgical path through the opticocarotid and carotid-oculomotor triangles and allowing the decompression and safe mobilization of the optic nerve [3-5]. Two main techniques have been used, the extradural and the intradural AC [6]. Intradural AC was the preferred technique before 1980 while extradural AC was popularized by Dolenc in 1985 [7-8]. The preference for intradural or extradural AC is a matter of debate, and experts vary in their opinion, often guided by the surgeon’s preference [9]. Despite the several advantages and limitations described for each technique, there is no evidence to support a technical preference¨[6,9]. However, there is an increasing demand to adapt surgical corridors to individual relevant anatomy and disease, and several technical advancements and operative modifications have been introduced for tailored and complete AC [1,9-10].

The aim of this study is to present and discuss the tailored AC concept through illustrative cases.

## Case presentation

This study is a retrospective record review of three patients who underwent AC for the treatment of parasellar pathologies that involved the ACP and optic canal. Clinical and surgical records, ophthalmological examinations, which included computerized campimetry, radiological data as magnetic resonance imaging (MRI), computed tomography (CT) scan, and CT angiography, AC technique applied, and outcome were reviewed. A written informed consent form was obtained from all patients. A review of the relevant literature on AC was performed in the PubMed, LILACS, and SciELO databases.

Case 01

Presentation

**Figure 1:**
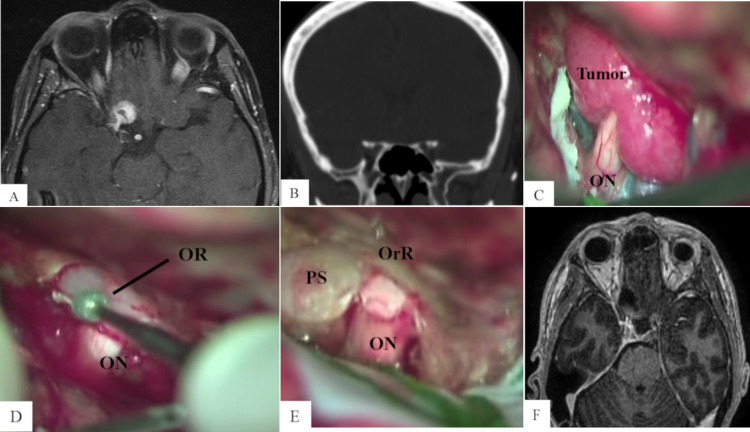
Tailored intradural AC (optic canal unroofing) (A) Preoperative contrast-enhanced axial T1-weighted MRI shows a small type III anterior clinoidal meningioma on the right side extending into the optic canal. (B) Preoperative coronal bone window CT scan does not show hyperostosis of the ACP. (C) Tumor involving the optic nerve and its attachment to the ACP. (D) The tumor was removed and the dura over the roof of the optic canal was cut, preparing for optic canal unroofing with a 2-mm high-speed diamond drill. (E) A small piece of the tumor was removed from the optic canal and gross total resection was achieved. (F) Postoperative contrast-enhanced axial T1-weighted MRI shows total tumor resection. ON, optic nerve; OR, optic roof; PS, planum sphenoidale; OrR, orbital roof; CT, clinoid tip

A 47-year-old female patient presented with gradual visual loss in the right eye over the last four months. Computerized campimetry revealed nasal hemianopia and superior temporal quadrantanopia in the right eye. Contrast-enhanced MRI showed a type III anterior clinoidal meningioma involving the optic nerve and extending into the optic canal (Figure 1, panel A). CT scan did not reveal tumor-associated hyperostosis (Figure 1, panel B).

Description of Technique

The patient was positioned in dorsal decubitus. The head was fixed in a three-pin device, elevated, turned 30 degrees to the contralateral side, and slightly extended. A pterional craniotomy was performed and the dura mater was opened. The Sylvian fissure was dissected and the frontal lobe gently retracted. The tumor was identified involving and compressing the right optic nerve (Figure 1, panel C). The tumor was then removed with bipolar coagulation, microscissors, and ultrasonic aspirator. A small piece of tumor was identified, extending into the optic canal. The optic canal unroofing (tailored technique) was performed using a small diamond drill bit with continuous irrigation (Figure 1, panel D). The remaining tumor was then safely removed within the optic canal and total resection was achieved (Figure 1, panel E).

Outcome

The patient had a good outcome with visual improvement during the follow-up. Contrast-enhanced MRI showed total resection (Figure 1, panel E).

Case 02

Presentation

**Figure 2:**
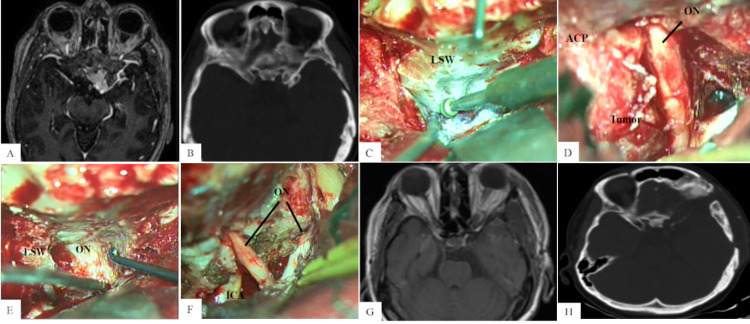
Hybrid technique for complete AC (A) Preoperative contrast-enhanced axial T1-weighted MRI shows a complex type II anterior clinoidal meningioma on the left side extending into the optic canal. (B) Preoperative axial bone window CT scan shows large hyperostosis of the ACP. (C) First step: extradural drilling of the sphenoid ridge. (D) Second step: intradural exploration showing the optic nerve compressed by the tumor and the hyperostotic ACP. (E) Third step: proceeding with total extradural AC. (F) Gross total resection of the tumor and decompression of the optic nerve is achieved. (G) Postoperative contrast-enhanced axial T1-weighted MRI shows no residual tumor. (H) Postoperative axial bone window CT scan shows total AC. AC, anterior clinoidectomy; LSW, lesser sphenoid wing; ACP, anterior clinoid process; ON, optic nerve; ICA, internal carotid artery

A 63-year-old female patient presented with progressive visual loss in the left eye and facial hypoesthesia on the same side over the last seven months due to recurrent anterior clinoidal meningioma. The preoperative MRI in 2012, before the first surgery, revealed an extra-axial lesion occupying the opticocarotid cistern with homogeneous contrast enhancement and dural attachment in the superolateral aspect of the ACP suggestive of type II anterior clinoidal meningioma, with minimal hyperostosis of the ACP on bone window CT scan. During the first surgery, the soft portion of the tumor was removed, but not the bone infiltration. Histopathological examination confirmed meningioma grade I (World Health Organization). Six years later, the MRI and CT scan showed intradural tumor recurrence with encasement of the ICA and extensive hyperostosis of the sphenoid wing and ACP (Figure 2, panels A and B). Neurological examination showed trigeminal neuropathy and visual loss, confirmed by computerized campimetry as a central scotoma.

Description of Technique

The patient was positioned in dorsal decubitus with an ipsilateral shoulder roll. The head was fixed in a three-pin device, elevated, turned 30 degrees to the contralateral side, and slightly extended. A pterional approach was performed. The sphenoid ridge was drilled and the meningo-orbital band was carefully cut to expose the ACP. Several anatomic bone changes were observed. Extensive extradural drilling of the lesser and greater sphenoid wing with a high-speed drill was performed (Figure 2, panel C). Then, the dura was opened and the soft portion of the tumor and the hyperostotic ACP were identified (Figure 2, panel D). After the intradural inspection, a total extradural AC was carried out to optimize optic nerve decompression (Figure 2, panel E). The falciform ligament was opened and free mobilization of the optic nerve was possible, followed by tumor removal. A gross total resection and removal of the hyperostotic ACP were achieved (Figure 2, panel F).

Outcome

The patient had a good outcome with visual improvement during the two-year follow-up. Contrast-enhanced MRI showed no residual tumor (Figure 2, panel G). CT scan revealed total clinoidectomy (Figure 2, panel H).

Case 03

Case Presentation

**Figure 3:**
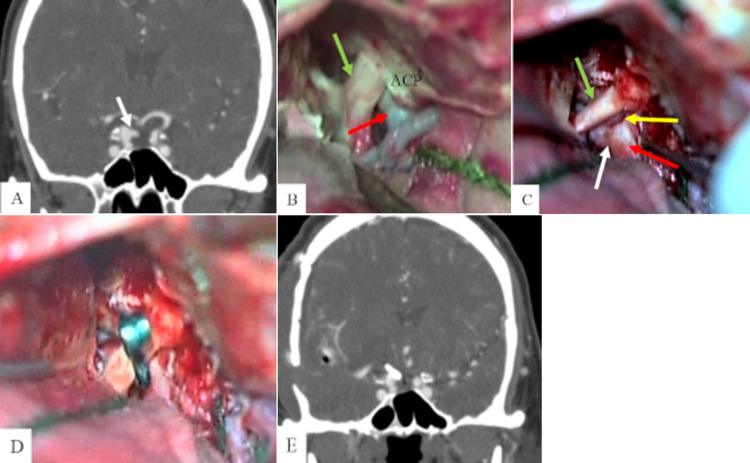
Tailored intradural AC (partial resection) (A) Preoperative coronal CT angiography reveals a 7-mm saccular aneurysm of the ICA, ophthalmic segment, on the right side (white arrow). (B) The intradural view shows the optic nerve (green arrow), ICA (red arrow), and ACP, but not the aneurysm. The aneurysm and the ophthalmic artery cannot be fully visualized due to the ACP. (C) The aneurysm and its neck (white arrow), as well as the ophthalmic artery (yellow arrow), are clearly identified after partial AC (optic canal unroofing and ACP tip osteotomy). (D) The surgical clip is successfully applied and the aneurysm is occluded, with good control of the ophthalmic artery. (E) Postoperative coronal CT angiography shows total occlusion of the aneurysm. ACP, anterior clinoid process; ICA, internal carotid artery; AC: anterior clinoidectomy

A 62-year-old female patient presenting with recurrent scintillating scotoma in the right eye about 12 months ago. CT angiography revealed a 7-mm saccular paraclinoid aneurysm of the right ICA, with the neck measuring 3.5 mm (Figure 3, panel A). The neurological exam was normal and computerized campimetry did not reveal any visual impairment.

Description of Technique

The patient was positioned in dorsal decubitus with an ipsilateral shoulder roll. The head was fixed in a three-pin device, elevated, turned 30 degrees to the contralateral side, and slightly extended. The standard pterional approach and durotomy were performed. The Sylvian fissure was opened and the optic nerve, ICA, and ACP were exposed. Until this step, a microscopic inspection was not able to view the aneurysm neck (Figure 3, panel B). Scalpel durotomy extending from the planum sphenoidale to the lateral aspect of the ACP tip was performed. A tailored intradural AC was carried out with optic canal unroofing and ACP tip osteotomy using a 2-mm high-speed diamond drill bit. The falciform ligament was opened, the optic nerve was gently mobilized, and the neck of the aneurysm and the ophthalmic artery were clearly identified (Figure 3, panel C). An aneurysm clip was applied, with good control of the ophthalmic artery (Figure 3, panel D). Intraoperative fluorescein video angiography revealed complete aneurysm occlusion.

Outcome

The patient's visual function was kept intact and postoperative CT angiography revealed complete aneurysm occlusion (Figure 3, panel E).

## Discussion

The AC was registered for the first time over 70 years ago using the intradural AC technique [11]. In 1985, Dolenc published an article about the combined approach as a path to the treatment of carotid ophthalmic aneurysms and then popularized the extradural technique [5,7]. Since then, several authors have been describing new techniques of AC for many parasellar lesions [3,7,8,11-14].

The most frequent approach for AC reported in the literature is the pterional approach [10]. However, many approaches have also been described to access the ACP, including orbitozygomatic craniotomy, lateral supraorbital approach, and, more recently, the endoscopic endonasal approach [1,10,15-17].

AC is a key microsurgical step for the safe and successful management of paraclinoid aneurysms and parasellar neoplastic lesions, increasing the surgical path through the opticocarotid and carotid-oculomotor triangles and allowing decompression and safe mobilization of the optic nerve [3-5]. Two main techniques have been used, the extradural and the intradural AC [11]. The surgical landmarks change according to the technique of AC [1,4,11]. Extradural AC has been reported as a technically demanding technique that requires precise knowledge of the 3-D anatomy of the ACP and its neighborhood [18]. However, the preference for intradural or extradural AC is a matter of debate, and experts vary in their opinion, often guided by the surgeon’s preference [9]. More recently, the hybrid method has been described as a versatile method to combine the advantages of the intradural and extradural techniques [6,8]. Hybrid techniques start with intradural visualization of the optic nerve and ICA, followed by extradural AC [9]. They can increase the safety of AC by providing easily identifiable landmarks and reducing intradural bone drilling, which could put the adjacent neurovascular structures at risk [8]. The hybrid technique can be useful for complex lesions with important anatomical deformation [3,6-7,10,19].

Intradural and extradural techniques are widely accepted, each one with relative advantages and disadvantages [6]. The main advantage of the intradural technique is having all adjacent neurovascular structures under visual control while drilling, crucial for clipping aneurysms of the ophthalmic segment of the ICA, allowing protection of the aneurysm dome, which can erode the ACP body [1,9,11]. Intradural AC also provides exposure of the optic nerve and proximal ICA without entering the cavernous sinus [7,14]. On the other hand, injury to the neurovascular structures is one of the most feared risks during drilling; even a small slip of the drill can lead to catastrophic situations [9,13,20].

The main advantage of the extradural technique is that it is entirely performed in the extradural space, allowing a layer of the dura to protect the adjacent eloquent structures and to avoid subarachnoid spreading of bone dust and postoperative headache [6]. Additionally, the extradural AC allows the possibility of extensive bone removal as compared to the intradural technique [19,21]. However, the extradural AC entails peeling the temporal dura propria from the lateral wall of the cavernous sinus along the entire length of the ACP, which can notoriously cause venous bleeding and/or potential damage to the nerves traveling in the sinus and superior orbital fissure [6].

ACP is generally composed of compact bone and is involved by clinoidal ligaments and dural elements, however, pneumatized ACPs are observed in up to 28% of the patients [4,7]. Intense pneumatization of the sphenoid bone can limit AC due to the high risk of sphenoid sinus opening and, ultimately, cerebrospinal fluid leak [22]. In such situations, bone removal should be minimized to a sufficient extent, not adequate for the desired anatomical exposure of target structures [5,11]. Ossification of the clinoidal ligaments are less frequent but can be noted in both the caroticoclinoid and interclinoid ligaments in approximately 17% and 2.8% of the patients, respectively [4]. Ossification of the clinoidal ligaments can significantly contribute to a change in the surgical strategy and generally requires a tailored AC to avoid injury to neurovascular structures, especially in cases of the caroticoclinoid foramen [23]. Besides anatomical reasons, AC can also be limited by the lesion itself. En-plaque meningiomas with significant ACP hypertrophy make the extradural AC too demanding, placing the optic and oculomotor nerves at risk [1]. Furthermore, paraclinoid aneurysms that erode the ACP can also limit the extradural AC [14]. In such cases, intradural is preferred over extradural AC with cervical carotid artery control [24]. Nevertheless, a preoperative thin-slice CT scan with 3D reconstruction is essential for an adequate understanding of the ACP anatomy as a pneumatization pattern, ligament ossification, hypertrophy, or even bone erosion [3,23,25].

An intradural AC is indicated for small and soft intradural lesions around the ACP, such as type III anterior clinoidal meningioma or carotid-ophthalmic aneurysms, or when optic nerve mobilization is mandatory for greater exposure of the opticocarotid and carotid-oculomotor triangles to access lesions that extend to the upper clivus and interpeduncular fossa [5,7,11-12]. The incision of the optic nerve sheath and the distal dural ring facilitates the mobilization of the optic nerve and promotes wide exposure and access around the ICA to remove parasellar and suprasellar tumors [22]. Intradural removal of the ACP with fracture of the optic strut requires minimal drilling, resulting in a decreased risk of injury to the optic nerve and a shortened time for clinoidectomy, known as the “en bloc” technique [12,14]. There are several techniques for intradural AC based on the type of lesion and surgeon’s experience [8,11-14].

Extradural AC is commonly preferred for complex lesions of the lesser sphenoid wing and cranial nerve compression that cross through the superior orbital fissure and is especially used in large parasellar meningiomas with extensive bone involvement, which promotes early tumor devascularization [5]. However, certain aspects become dangerous such as a caroticoclinoid bone ring and extensive aeration of the sphenoid bone [4]. Lesser sphenoid wing en plaque meningiomas with ocular proptosis usually require extradural AC and orbital roof resection [24-26]. In cases of small type III anterior clinoidal meningioma, the extradural AC technique provides an exaggerated bone exposure and high risk of neurovascular injuries [1,3-4,27-29].

Beyond the scope of the intradural and extradural techniques, the concept of tailored AC has been developed to balance the risks of total removal and to avoid more extensive and time-consuming techniques. It has been noted that ACP does not need total removal in all cases, and custom-tailored AC can be practiced to avoid potential mechanical and thermal complications [10,13,20,30]. The choice of tailored AC is related to the size and location of the lesion, aiming to remove just enough bone to treat the lesion [10]. Several technical advancements and operative modifications have been introduced for tailored AC [9]. The ACP can be removed partially or totally by both extradural and intradural techniques, however, the extradural one nearly always achieves complete clinoidectomy [9,11]. The amount of bone removal has been classified as minimal (tip of the ACP), partial (tip and head of ACP), subtotal (tip, head, and body of the ACP), and total (tip, head, body, and base of the ACP) [10]. For carotid-ophthalmic aneurysms, intradural anterior clinoid tip removal may be sufficient to expose the microsurgical field and proximal control of the artery, as well as for small meningiomas of the optic foramen, the intradural optic canal unroofing, and the opening of the falciform ligament may be sufficient for complete removal of the tumor and its dural attachment [10,23].

Lastly, skull base surgery has been modified in an attempt to make it less morbid [28-29]. Tailored AC aims to provide adequate exposure with less risk of neurovascular injury, allowing enough space to safely treat parasellar lesions. The type, size, and location of the lesion, patient's anatomy, as well as surgeon’s experience, should always be considered for surgical planning.

## Conclusions

Through three illustrative cases, we demonstrated that the "tailored AC " aims to provide adequate exposure with less risk of neurovascular injury, allowing enough space to safely treat parasellar lesions. The type, size, and location of the lesion, as well as the surgeon’s experience, should always be considered for surgical planning.
